# Secondary use of patient data within decentralized studies using the example of rare diseases in Germany: A data scientist's exploration of process and lessons learned

**DOI:** 10.1177/20552076241265219

**Published:** 2024-08-10

**Authors:** Michele Zoch, Christian Gierschner, Anne-Katrin Andreeff, Elisa Henke, Martin Sedlmayr, Gabriele Müller, Jenny Tippmann, Helge Hebestreit, Daniela Choukair, Georg F. Hoffmann, Fleur Fritz-Kebede, Nicole Toepfner, Reinhard Berner, Stephanie Biergans, Raphael Verbücheln, Jannik Schaaf, Julia Fleck, Felix Nikolaus Wirth, Josef Schepers, Fabian Prasser

**Affiliations:** 1 Institute for Medical Informatics and Biometry, Faculty of Medicine and University Hospital Carl Gustav Carus, TUD Dresden University of Technology, Dresden, Germany; 2 Center for Evidence-based Healthcare, Faculty of Medicine and University Hospital Carl Gustav Carus, TUD Dresden University of Technology, Dresden, Germany; 3 Thiem-Research GmbH, Cottbus, Germany; 4 Center for Rare Diseases – Reference Center Northern Bavaria, University Hospital, Julius-Maximilians University, Würzburg, Germany; 5 Center for Rare Diseases, University Hospital Heidelberg, Heidelberg, Germany; 6 University Centre for Rare Diseases and Department of Pediatrics, Faculty of Medicine and University Hospital Carl Gustav Carus, TUD Dresden University of Technology, Dresden, Germany; 7 Medical Data Integration Center (meDIC), 27203 University Hospital Tübingen, Tübingen, Germany; 8 Institute of Medical Informatics, 9173 Goethe University Frankfurt, University Hospital, Frankfurt, Germany; 9 Center for Rare Diseases, 9165 RWTH Aachen University Hospital, Aachen, Germany; 10 Berlin Institute of Health (BIH) at Charité Universitätsmedizin Berlin, Berlin, Germany

**Keywords:** Data management, decentralized study, FHIR, interoperability, secondary use of patient data, rare diseases

## Abstract

**Objective:**

Unlocking the potential of routine medical data for clinical research requires the analysis of data from multiple healthcare institutions. However, according to German data protection regulations, data can often not leave the individual institutions and decentralized approaches are needed. Decentralized studies face challenges regarding coordination, technical infrastructure, interoperability and regulatory compliance. Rare diseases are an important prototype research focus for decentralized data analyses, as patients are rare by definition and adequate cohort sizes can only be reached if data from multiple sites is combined.

**Methods:**

Within the project “Collaboration on Rare Diseases”, decentralized studies focusing on four rare diseases (cystic fibrosis, phenylketonuria, Kawasaki disease, multisystem inflammatory syndrome in children) were conducted at 17 German university hospitals. Therefore, a data management process for decentralized studies was developed by an interdisciplinary team of experts from medicine, public health and data science. Along the process, lessons learned were formulated and discussed.

**Results:**

The process consists of eight steps and includes sub-processes for the definition of medical use cases, script development and data management. The lessons learned include on the one hand the organization and administration of the studies (collaboration of experts, use of standardized forms and publication of project information), and on the other hand the development of scripts and analysis (dependency on the database, use of standards and open source tools, feedback loops, anonymization).

**Conclusions:**

This work captures central challenges and describes possible solutions and can hence serve as a solid basis for the implementation and conduction of similar decentralized studies.

## Introduction

The continuous development and improvement of technologies and data analysis methods have had a significant impact on healthcare in recent years.

These include the secondary use of data, which was primarily collected for another purpose^1^ (e.g. documentation of care, billing in the hospital information system), for medical studies. Secondary use of data opens up the possibility for the generation of new data-driven medical insights and is a viable alternative to time-consuming and cost-intensive clinical studies. For example, data collected for documentation and billing in the hospital information system can be used for analyses in the context of a medical study.^2^

**Figure 1.**
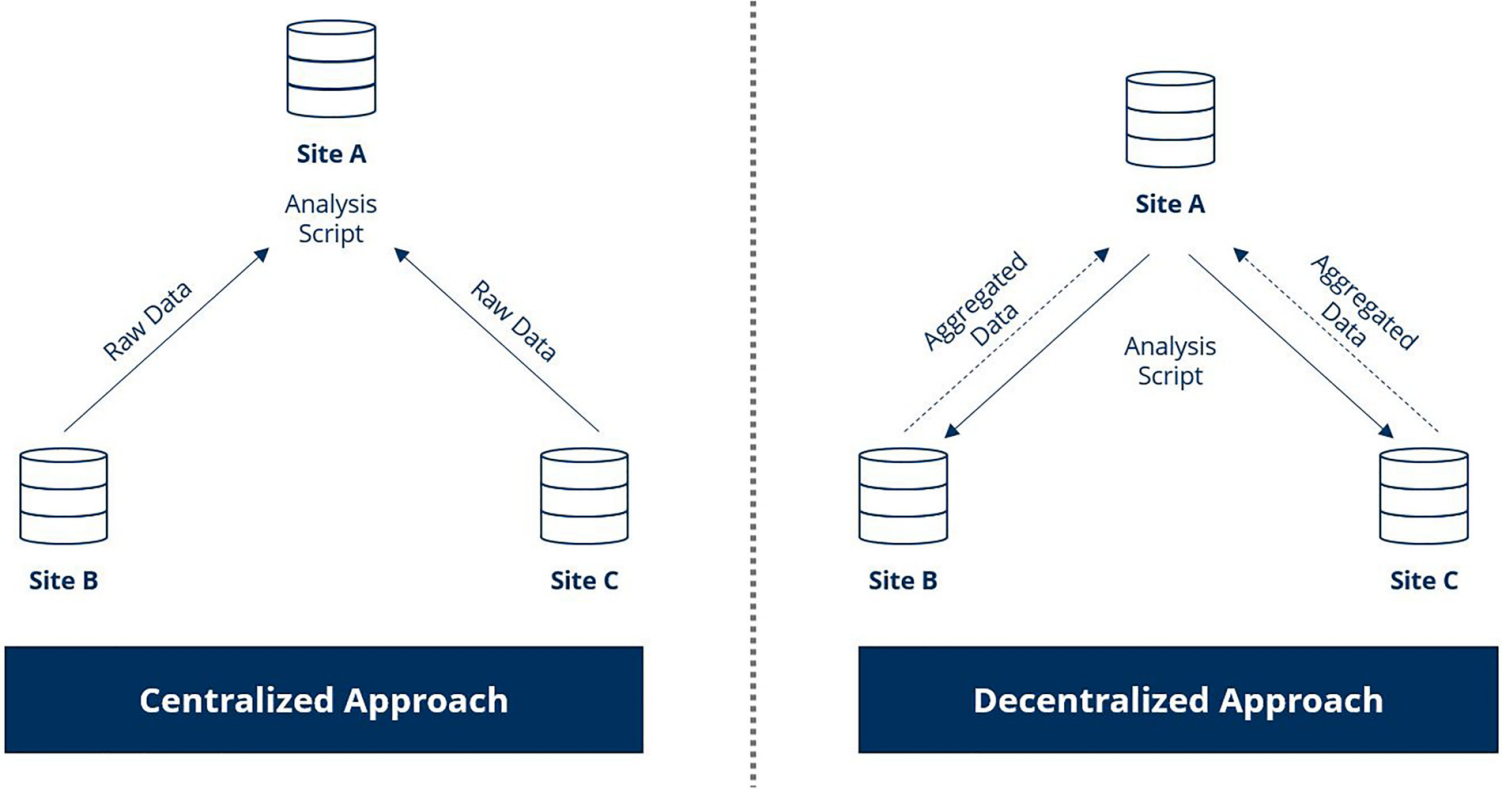
Centralized vs. decentralized analysis.

Within the framework of secondary use of patient data, centralized or decentralized studies can be conducted (see Figure 1). They refer to research models in which data collection and decision-making are conducted across multiple locations. In the centralized approach, raw or pseudonymized data from different sites is transferred to a trusted location. This location (see Figure 1, site A) aggregates the data and executes the analysis scripts to generate results. In contrast, with the decentralized approach, the data is analyzed locally by running centrally designed analysis scripts at each participating institution and returning only the (anonymous) statistical results. As a consequence, sensitive patient data remains at the sites but can still be used for research in a privacy-preserving manner.^3^

Decentralized studies based on secondary use of patient data offer great potential, especially for rare diseases. As the patients are rare by definition, it is necessary to aggregate the data from as many study participants as possible in compliance with data protection principles. The principles are upheld if the patients cannot be identified, i.e. remain anonymous. By merging only aggregated data, which contains the results of the analysis scripts and not the actual patient data, it is not possible to identify individuals. An as of yet unused potential exists for data science and privacy-preserving methods to improve medical decision-making and to minimize the need for expensive clinical trials.

While decentralized studies based on secondary use of patient data offer several benefits, such as enlarged pool of potential participants, increased diversity in participation due to widespread geographical involvement and absence of direct patient engagement,^4^ they also face certain challenges.

- **Management**: Effectively managing a study with multiple sites requires seamless coordination, communication and monitoring to ensure smooth operations and consistent data collection. This necessitates the establishment of an overarching monitoring mechanism.^5^
- **Infrastructure**: An essential component for decentralized data collection, storage and analysis is a secure and functional technological infrastructure.^3^
- **Interoperability**: Due to different data sources, methods for data integration and data modeling, the semantics and syntax of the required data may vary greatly at the different locations. To ensure high data quality, interoperability – particularly through the adherence of standards – must be guaranteed.
- **Privacy**: Protecting sensitive patient data is of utmost importance, and implementing approaches such as federated data sharing and privacy-preserving infrastructures is essential. In the past, these methods have been sporadically utilized in medical studies, but are not in widespread use yet.^3^
- **Regulatory compliance**: Ensuring regulatory compliance at all study sites is critical to uphold research integrity. The federal structure of Germany poses a significant challenge in achieving this. For example, there are different interpretations of current data protection legislation per state or different ethics committee requirements at study sites.

To meet these challenges, decentralized studies need to be designed and conducted with a strong focus on quality. The aim of this paper is to describe the process ensuring high-quality decentralized studies, by pinpointing the lessons learned while conducting research on rare diseases in Germany.

## State of the art

### Medical Informatics Initiative: German research infrastructure

The German Medical Informatics Initiative (MII) aimed to bring significant enhancements in medical research and healthcare in Germany by harnessing digital technologies and data, and by devising innovative solutions to health-related challenges. Supported by the German Federal Ministry of Education and Research, all university hospitals in Germany have received funding to advance the objectives of the MII.^6,7^ These objectives encompass the preparation of data for medical decision support and scientific research, and the reinforcement of Germany's position as a scientific hub in the domain of Clinical Data Science. Although the four consortia – DIFUTURE,^8^ HiGHmed,^9^ MIRACUM^10^ and SMITH^11^ – adopted distinct approaches, they form a common infrastructure based on the following key components:

- Each university hospital established a **data integration center** for the pooling and processing of patient data from clinical care. These data are made available for medical research in a privacy-compliant manner.
- All consortia pledged their commitment to the **Core Data Set** (CDS) **of the Medical Informatics Initiative** (MII-CDS). The MII-CDS is a data and information model divided into base modules and extension modules^12,13^: The base modules include general medical concepts, such as “Person” and “Diagnosis”; the extension modules define data of specific specialty areas, such as “Oncology” or “Molecular Genetic”. The modules are specified using profiles of Health Level 7 (HL 7) Fast Healthcare Interoperability Resources (FHIR) and explained in more detail by Unified Modelling Language (UML) diagrams and implementation guides. The uniform definition of data sets across all data integration centers ensures interoperability and thus serves as a basis for collaborative data analyses.
- The **German Portal for Medical Research Data** (German: “Forschungsdatenportal für Gesundheit” (FDPG)^14^) is the central point of contact for data requests from German university hospitals for medical research projects.^15^ Feasibility queries serve as a real-time overview for researchers of the number of existing cases in the data integration centers based on search criteria.^16^
- To request research data through the portal, a nine-step process must be followed^17^: (1) registration in FDPG, (2) feasibility request, (3) application for data provision or distributed analysis, (4) decision of the Use and Access Committee (UAC) at each site, (5) data use contract, (6) publication in the FDPG project registry, (7) use of the requested data or analysis results, (8) publication of the results in FDPG, (9) listing of the scientific publications in FDPG.
- At each local site, the **UAC**, which is usually composed of representatives from the board of directors of the university hospital, data protection offices, clinicians, biometrics / epidemiologists, is responsible for the decision on provision of data or analysis results. The UAC evaluates the request according to organizational, (data protection) legal and scientific aspects and decides on the participation of its own institution.
- A **transfer office** is an intermediate point between the data integration center and the scientists who request and use data. It is responsible for accepting requests for use and initiating the respective internal process of reviewing the research projects and decide on participation. After evaluation of the UAC, it is making the requested data available.
- The provision of data or analysis results is done by the **data management center**. The data management center is an institution, which is responsible for the preparation and execution of the data transfer as well as for the preparation of the data and sending it to the data recipients.
- Analysis scripts are required to conduct distributed analyses. These can be developed by either the researcher or the data management center and must be based on the MII-CDS.
- The data use request and contract based on the standardized form of the MII are concluded and the developed scripts are distributed.

To test the established infrastructure and the developed methods, cross-consortium use cases were initiated. One of these use cases was “Collaboration on Rare Diseases” (CORD-MI), which focuses explicitly on the field of rare diseases.

### CORD-MI: Retrospective studies on the example of four rare diseases

In the European Union, a disease is considered rare if it affects less than 1 in 2000 people;^18–20^ in the United States, if less than 200,000 people are affected. It is assumed that there are more than 7000 distinct clinical entities^18,21,22^ and that approximately 3.5–5.9% of the world population, i.e. approximately 263–446 million people worldwide, suffer from a rare disease.^23^ Rare diseases have in common that they are often life-threatening or have a chronic course with negative effects on the quality of life and life expectancy.^24,25^ They represent major challenges for patients and their families as well as for the health care system^20,21^ due to accompanied high morbidity. Consequently, the healthcare utilization of children and adults with rare diseases causes enormous costs.^26^

CORD-MI, which involved a total of 24 German university hospitals, pursued four goals: (1) improving the visibility of rare diseases, (2) providing insights into the reality of care, (3) improving the quality of patient care and (4) strengthening research in the field of rare diseases.^27^ As part of this, decentralized studies were defined and conducted in the project network.

Different rare diseases were selected as prototypes for those decentralized studies in order to take into account both the spectrum of different disease patterns and the spectrum of different data (also with regard to availability, quality and modeling). The following diseases served as examples: (1) cystic fibrosis (CF), (2) phenylketonuria (PKU) and (3) Kawasaki disease and multisystem inflammatory syndrome in children (MIS-C).

1. **CF **is a rare congenital multi-organ disease.^28^ Health improvements have led to more pregnancies in women with CF, but these pregnancies carry an increased risk for both the mother and the child.^29^ Specialized centers, often located at university hospitals, are recommended for prenatal care and delivery in CF. The primary objective of this study was to determine the delivery rate of CF patients in the specialized centers in relation to all deliveries.
2. **PKU **is a rare congenital metabolic disease.^30^ Despite a significant number of patients being over 18 years old, there were hints that in adult medicine sufficient care pathways on PKU is lacking. Cross-sectional studies have indicated a clustering of relevant internal, neurological and psychiatric symptoms in adults with PKU,^31,32^ but larger or systematic studies are currently lacking.
3. **Kawasaki disease** is a systemic vasculitis in children aged 0–5 years.^33^ In recent years, more sensitive case definition and improved diagnosis have been demonstrated.^34^ The incidence of Kawasaki disease during the severe acute respiratory syndrome coronavirus type 2 (SARS-CoV-2) pandemic was significantly higher than comparable prior-year periods. However, it turned out that as a result of SARS-CoV-2 infections in children and adolescents, a new clinical picture had emerged that was similar to Kawasaki disease, but also had significant differences in its characteristics and clinical presentation.^35^ This “new” clinical picture was summarized under the acronym **MIS-C** (or synonym pediatric inflammatory multisystem syndrome) and is a rare inflammatory disease in children occurring after a SARS-CoV-2 infection. The different incidences should be investigated in the study.

## Method

As part of the project CORD, the process of decentralized studies had to adhere to the requirements of the MII and the aforementioned infrastructure. These requirements encompass both administrative and technical aspects. For example, privacy concepts and study protocols for legal and ethical review had to be described in detail, and administrative steps had to be followed, especially in the context of the FDPG. In addition, analysis scripts had to be developed based on FHIR profiles compliant with the MII-CDS; and the data management office had to take into account that the aggregation of the resulting data is done according to the previously defined data protection-compliant procedure as well as contributes to answering the research questions. Based on these requirements and a thematic grouping, specific procedural steps were formulated within expert group.

**Figure 2.**
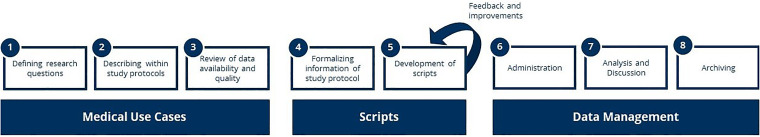
Coherent process (steps 1–7) for decentralized studies on the example of rare diseases in Germany.

For conducting the decentralized studies, an interdisciplinary team of experts in medicine, public health and data science developed and went through a process, which is shown in Figure 2. The eight steps, thematically bundled into three categories, are described below.

We conducted retrospective, observational, descriptive studies. These decentralized studies involved multiple sites (cross-sectional). Data from the clinical information systems (prepared by the data integration centers) was used. This data was gathered for documenting patient conditions, medical interventions and facilitating healthcare service billing. The dataset encompasses various elements such as demographic details, diagnoses, procedures, laboratory results and more. The studies were part of the three-year CORD-MI project and were conducted within 13 months (18 November 2022 to 15 December 2023).

### Medical use cases

The decentralized studies served to answer medical questions (*step 1*). The research questions of the medical use cases, which were specified in detail by primary and secondary questions, are listed in Table 1.

**Table 1.** Research questions of the medical use cases.

Medical Use Cases	Primary Research Questions	Secondary Research Questions
(1) CF and pregnancy or delivery	(a) What was the percentage of pregnant women with CF who gave birth in a hospital with a CF center?	(b) How common were complications during these pregnancies and childbirths? (c) Which outcomes were observed in these children?
(2) PKU, comorbidities in adulthood and pregnancy or delivery	(a) How often were internal, neurological and psychiatric diseases documented in combination with PKU?	(b) Did internal, neurological and psychiatric diseases in PKU patients depend on age? (c) How common were complications of PKU during pregnancy and childbirth? (d) Which outcomes were observed in the children of PKU women?
(3) Kawasaki disease and MIS-C	(a) How often were Kawasaki disease or MIS-C documented over time?	(b) How often were the diagnoses of Kawasaki disease or MIS-C documented in combination with COVID-19? (c) Did the frequency of Kawasaki disease or MIS-C diagnosis differ regionally in Germany? (d) Did the incidences of Kawasaki disease or MIS-C differ between genders and age groups?

CF: cystic fibrosis; PKU: phenylketonuria; MIS-C: multisystem inflammatory syndrome in children; COVID-19: coronavirus disease 2019.

Medical experts, together with experts in public health, wrote study protocols (*step 2*) describing the study aims, methods and case definition, eligibility criteria, study variables as well as legal and ethical considerations. Based on those study protocols, the team formulated an overarching data protection concept. The study protocols (*step 2*) are available on Zenodo (10.5281/zenodo.10656436^36^). Individual sites made adjustments to the study protocols according to local ethics committee requirements. Local data protection concepts and local data protection impact assessments have also been formulated. These documents are of great value in the context of sharing anonymous results data, as they serve to mitigate privacy concerns and ensure that data is non-personal in the legal sense.

Before conducting a study, it is beneficial to review the quality of the underlying data (*step 3*). The requirements for sensitivity and specificity of the inclusion and exclusion criteria are particularly high for rare diseases, as incorrect assignments to the study population in small patient collectives could have a strong impact in the subsequent analyses. Therefore, we initially checked the inclusion and exclusion criteria by having the patient cohorts found in selected centers compared with their documentation by the treating physicians. This process was repeated iteratively until both cohorts matched as closely as possible. Feasibility requests can also be helpful in this step of the process. A feasibility study guarantees a systematic assessment to determine the practicality and viability of the planned study.^37^ A feasibility request was waived because data storage and harmonization was part of the project CORD-MI itself.^38^ The individual data integration centers are responsible for ensuring data quality. There was also an additional use case for data quality monitoring in the overall project. To guarantee the accuracy of the outcomes, interim results needed validation from medical professionals. These experts carefully reviewed each case to evaluate plausibility and reliability of the data and results.

### Scripts

Data scientists formalized the information from the study protocols to both define the target set of scripts to be developed and they determined the data elements needed (*step 4*). The results are data descriptions that list the individual data items, particularly in relation to the required terminologies and classifications. These can be found on Zenodo (10.5281/zenodo.10213532^39^).

An interdisciplinary team developed the scripts and discussed the development, received feedback as well as performed adjustments and improvements. The scripts were developed to process the MII-CDS using R (*step 5*). The R Package fhirckrackr was used to download and flatten the FHIR data.^40^ Thus, the hierarchical structure of FHIR (usually encoded in JSON or XML) could be broken up to transform it into tabular data.

To protect privacy in the resulting statistical tables, cell suppression with a frequency threshold rule with threshold 5 has been implemented.^41^ Thus, (absolute) results larger than zero and smaller than five were masked with the category “<5”. Given the implementation of the frequency threshold rule and the exclusive transmission of anonymous results data from the sites for analysis, patient consent was unnecessary.

The scripts are available in a separate Git repository (*step 5*): (1) CF and pregnancy or delivery,^42^ (2) PKU, comorbidities and pregnancy or delivery^43^ as well as (3) Kawasaki disease and MIS-C.^44^

### Data management

The medical use cases were defined and submitted individually as projects to the FDPG and published on the FDPG website (*step 6*): (1) CF and pregnancy or delivery, (2) PKU, comorbidities and pregnancy or delivery as well as (3) Kawasaki disease and MIS-C (for permanent export of the websites and an English translation see Zenodo (10.5281/zenodo.11034586^45^).

The information (including project goal, project description and required data) was forwarded to the transfer offices of the data integration centers (*step 6*). There, the UAC individually decided on the provision of the analysis results.

The individual study sites executed the scripts and provided the aggregated data to the data management center (*step 7*). For the three use cases, the data management center provided the infrastructure to store the data securely by providing password-protected folders for each individual site on a cloud. They merged the data from the study sites and prepared it for the medical experts according to research questions. The data was then sent to the data recipients (i.e. the medical experts). Medical experts analyzed the results (*step 7*) for separate publication.

The data from the three medical use cases – both at the individual sites and the aggregated data for the data recipients – were forwarded to the data recipients / medical experts and archived (*step 8*). Other elements important to the research project, such as the scripts, were also archived. The individual sites and the aggregated data were archived in a structured file repository of data management center. The following additional metadata was specified: Identifier, project title, description, applicant, date of data provision, contact applicant, information of publications (title, doi, authors), rights of subsequent use and keywords.

### Formulation of lessons learned

Looking back on the project, the interdisciplinary team of experts in medicine, public health and data science formulated the most impressive lessons learned for them.

## Results

By following the process described in the Methods section, we were able to extract, analyze and aggregate data for the three use cases. 17 sites participated in the first two studies of (1) CF and pregnancy or delivery and (2) PKU, comorbidities and pregnancy or delivery; 14 sites participated in the third study of (3) Kawasaki disease and MIS-C.

### Lessons learned

Along the process, the following lessons were learned:

- (LL1) Collaboration of experts in medicine, public health and data science;
- (LL2) Attention to the database (existence of data);
- (LL3) Use of standards;
- (LL4) Use of open source tools;
- (LL5) Iterative process with feedback loops;
- (LL6) Implementation of frequency threshold rule;
- (LL7) Use of standardized forms;
- (LL8) Publicly available information on the project.

(LL1): For the seamless conduction of decentralized studies, collaboration between experts is crucial. This requires a high level of understanding of each other's professions (e.g. regarding the use of technical terms or the level of detail of the information provided). By sharing a common vision and goal – to improve research and care for patients with rare diseases – all participants were able to contribute their experience to expand the common knowledge.

(LL2): Understanding the origin of the data is equally crucial. Secondary data research involves the use of data that was primarily collected for a different purpose. For example, billing-related data from hospital information systems – prepared by data integration centers – was used for the studies described. The “Health Care Process Bias”,^46^ referring to the potential distortion of medical reality within data resulting from billing guidelines, poses several challenges. Consequently, it is important to consider this factor when structuring decentralized studies reliant on secondary use of patient data. Consequently, we could not use the International Statistical Classification of Diseases and Related Health Problems in the 10th Version (ICD-10-GM) codes on a case basis, but needed to define a patient with a rare disease on a patient-basis, integrating information from several in-patient stays and outpatient visits. Also, not all study sites were able to answer all research questions. For example, only 9 of 14 sites were able to provide information on the research question “Does the frequency of Kawasaki Disease or MIS-C differ regionally in Germany?” (see Table 1, research question 3c). This is due to the fact that not all data integration centers were able or allowed to integrate information about the zip codes of the addresses of their patients, although this information is part of the MII-CDS. Thus, the associated data protection concepts should be taken into account, when formulating the study protocol or developing the analysis scripts. On the one hand, it should be considered which data is available at all; and on the other hand, the required data elements should be described as precisely as possible in order to facilitate the later data provision. Likewise, the analysis methods – both in terms of data retrieval and statistical data evaluation – should be described in advance, in order to facilitate the later script development.

(LL3): The use of standards – terminologies and classifications as well as dataset descriptions and exchange formats – facilitates both the unique naming of data elements and the development of scripts. Although standards (especially FHIR) were used, the individual interpretation of each study site may vary. Thus, the development team discussed individual local peculiarities after trying to execute the scripts in many feedback loops. As a result, the scripts now take into account some exceptions. This was labor-intensive and time-consuming. In addition, the scripts had to be limited to ICD-10-GM, because the study sites have different levels of availability of ORPHAcodes. However, these are essential for unambiguous naming of the individual rare diseases.

(LL4): The use of open source tools – here R and fhircrackr – facilitates the collaborative development and delivery of the scripts. The use of open source tools should facilitate the execution of the decentralized study, as the study sites should have the scripts run in their own infrastructure. However, there are concerns and challenges with running third-party software. Some study sites performed all data management and analysis in an environment classified as critical infrastructure. Therefore, time-consuming IT-security approval processes were required, if novel open source software had to be used. For an individual study, it is challenging and not always feasible to invest the time and resources for these strict approval processes. This might cause individual study sites to require custom solutions or being unable to participate in an analysis.

(LL5): The iterative process allowed the scripts to be constantly improved. Thus, the study sites reported potential improvements or problems after tried execution. These were then discussed and resolved together. In this way, it was guaranteed that all study sites that wanted to participate in the studies were able to do so. Due to the large number of study sites, the feedback loops were very labor-intensive and time-consuming.

(LL6) The masking of small results (with “<5”) ensures privacy, but thus limits the interpretation of findings. Especially in the context of rare diseases, even small numbers of cases are important and can lead to a big difference. This means complex procedures and privacy-preserving methods,^47^ such as Secure Multi-Party Computation (SMPC), can be used. The latter refers to a set of cryptographic methods allowing the calculation of a mutual result between several parties without sharing the respective input data. One group of SMPC-methods are so-called “secure sum” protocols, in which data between three or more parties is aggregated without revealing the inputs of the single parties. Secure sum protocols are of particular use when researching rare diseases since the protocols can assist to increase sample sizes. Several tools support the practical execution of secure sum protocols (see e.g. EasySMPC^48^). However, the SMPC-methods only protect input data, while the data output of a SMPC-protocol still can contain personal data.

(LL7): Standardized forms were used to request and release data that were mutually agreed upon in the MII and accepted by each study site. They provide a secure legal framework for data use. Nevertheless, the bureaucratic effort was very time-consuming, which means that the contract process should be initiated early so that it does not become a showstopper. At every university hospital, individual ethics votes and votes of the UAC had to be obtained due to the individual data protection laws of the federal states and different regulations of the university hospitals. In future, a lead vote by a local ethics committee will suffice to speed up the process.

(LL8): A project has been created for all studies on the FPDG website. There, all interested parties can read about details and progress of the project. The publication of project information and protocols creates transparency.

## Discussion

We were able to successfully conduct decentralized studies on three medical use cases in the context of rare diseases on the basis of secondary use of patient data. Thus, the three studies are the first successfully performed studies in the context of the productive start-up of the FDPG in the German MII. For this purpose, we went through a process consisting of eight steps covering the definition of medical use cases and the development of scripts to data management, analysis and archiving. It led to results for all three different use cases, what is demonstrating the usability of the process in practice.

Furthermore, the successful implementation shows that secondary use of patient data can be used for investigating medical issues as part of decentralized studies. It successfully enabled the expansion of the data pool. However, limitations may be noted due to the divergent purposes of data collection and to the lack of data.

The process presented is aligned with the individual steps of an Observational Health Data Science and Informatics (OHDSI) study (study definition and design, review of data availability and quality, standardized analysis, study packages/script, execution, interpretation and write-up^49^). The research community successfully conducts a variety of different studies and has already gained a lot of experience in these regards. The sub-steps were bundled thematically. So far, the “Review of data availability and quality” was left out due to the project specifics. A query portal can be used to conduct feasibility studies on the data of German university hospitals.^16^

The presented process consists of eight steps, contributing to its perceived complexity. This complexity stems from bundling individual tasks according to the diverse responsibilities of experts from various domains, including medicine, public health and data science. The comprehensive description aims to facilitate the implementation of future studies. However, it is essential to consider automating or semi-automating specific steps to enhance efficiency. For instance, in data retrieval and evaluation (refer to *step 5* “Development of scripts”), parameterized scripts could offer a promising approach. Parts of scripts used in the presented studies, such as those for defining frequency threshold rules, have already been repurposed. Moving forward, it is crucial to investigate which aspects of the process can be generalized to solely require parameterized queries. Also, investigating whether there is a real reduction in conduct time and cost savings compared to clinical trials can be explored in future studies.

The lessons learned include both positive experiences and their successful implementation as well as potential for improvement. Numerous aspects extend beyond the scope of decentralized studies on secondary use of patient data in general and can be regarded as prerequisites for structured research endeavors. Thus, the collaboration between the experts (see LL1) should be continued and also raised to an international level. In this way, the data and knowledge pool can be continuously expanded. In order to utilize secondary use of patient data profitably and appropriately for research (see LL2), methods for harmonizing and structuring them must be evaluated. With regard to using interoperability standards (see LL3), it has to be checked if a combination with further tools, which especially increase the syntactic interoperability of the data, can be used to facilitate and speed up the process. For example, the complementary use of Observational Medical Outcomes Partnership (OMOP) Common Data Model (CDM) of OHDSI would be conceivable.^50,51^ The use of other international terminologies, such as Systematized Nomenclature of Medicine Clinical Trials (SNOMED CT), would be interesting, too, so that the data can also be compared and used internationally. Also, tools for the structured definition of data elements and their metadata, such as Portal of Medical Data Models (MDM)^52^ or ART-DECOR,^53^ can be beneficial here. Approaches need to be created to support coding in both medical practice and research as well as to assure the quality of data.

In addition, so far only structured data have been used as a basis; the use of unstructured data of text-based documentation, e.g. from physicians’ letters or free text information in a hospital information system, remains a challenge. Individual efforts (e.g.^54^) cannot yet be extended to the overall consortium. The data integration centers systematically investigate unstructured data by utilizing diverse tools and methodologies, such as by automating the indexing of medical texts. To minimize the concerns and challenges by using third-party software (see LL4), predetermined guidelines for the design and review of data analysis pipelines could help to streamline this process; as well as the development of standard solutions for certain analysis steps, which can then be pre-approved and used as components in various data analysis pipelines. For shortening the overall process and speeding up the feedback loops (see LL5) it needs to be examined whether standardized methods have potential to streamline the process. Tools, such as ATLAS^55^ from OHDSI, for the uniform definition of cohorts are conceivable. In addition, it should be checked to what extent methods can be applied to secure the output data as well (see LL6).

The use of standardized forms allowed for a legal framework to which each study site was committed (see LL7). Nevertheless, bureaucratic hurdles (e.g. customization study protocols for voting by the local ethics committee) still had to be overcome at the sites themselves. Here, a nationwide German solution would save a lot of time and resources. By publishing information about the studies (see LL8), the usefulness of the established structures of the MII and of the data analysis can be demonstrated for the society. This should be continued by also making the results of the studies publicly available.

## Conclusion

Despite the potential of decentralized studies, few papers have been published which focus on the implementation of this research model. Detailing the process and discussing lessons learned will help to better understand the characteristics of those kinds of studies. This will enable clinical and scientific researchers to design and conduct decentralized studies themselves.

We successfully conducted decentralized studies. The eight-step process shows the flow for research based on secondary use of patient data. In this way, medical knowledge could be acquired by using data already collected (i.e. within the clinic information systems), thus avoiding additional data collection.

The experience gained from the example of rare diseases can be applied to other disease entities. Above all, the collaboration between the different experts, the use of open source tools and standards and the establishment of feedback loops were beneficial.

In the future, it will be necessary to review how the individual process steps can be simplified and accelerated. For example, further exploration is needed to determine how cutting-edge approaches, particularly in dataset description and privacy prevention, can be integrated into the process.

## List of abbreviations

CF: Cystic fibrosis
CORD: Collaboration on Rare Diseases
DIFUTURE: Data Integration for Future Medicine
FDPG: German Portal for Medical Research Data (German: “Forschungsdatenportal für Gesundheit”)
FHIR: Fast Healthcare Interoperability Resource
HiGHmed: HiGHmed Medical Informatics
HL 7: Health Level 7
ICD-10-GM German: Modification of International Statistical Classification of Diseases and Related Health Problems in the 10th Version
LL: Lessons learned
MII: Medical Informatics Initiative
MII-CDS: Core Data Set of the Medical Informatics Initiative
MIRACUM: Medical Informatics in Research and Care in University Medicine
OHDSI: Observational Health Data Science and Informatics
OMOP CDM: Observational Medical Outcome Partnership Common Data Model
MIS-C: Multisystem inflammatory syndrome in children
PKU: Phenylketonuria
SARS-CoV-2: Severe acute respiratory syndrome coronavirus type 2
SMITH: Smart Medical Information Technology for Healthcare
SMPC: Secure Multi-Party Computation
SNOMED CT: Systematized Nomenclature of Medicine Clinical Trials
UAC: Use and Access Committee
UML: Unified Modelling Language
